# The Efficacy of High-Intensity Preoperative Physiotherapy Training on Postoperative Outcomes in Greek Patients Undergoing Total Knee Arthroplasty: A Quasi-Experimental Study

**DOI:** 10.7759/cureus.23191

**Published:** 2022-03-15

**Authors:** Dimitrios Vasileiadis, Georgios Drosos, Georgios Charitoudis, Ismene A Dontas, John Vlamis

**Affiliations:** 1 Laboratory for Research of the Musculoskeletal System, KAT General Hospital, Medical School, National and Kapodistrian University of Athens, Athens, GRC; 2 Department of Orthopaedic Surgery, University General Hospital of Alexandroupolis, School of Medicine, Democritus University of Thrace, Alexandroupolis, GRC; 3 Veterinary Medicine, Laboratory for Research of the Musculoskeletal System, KAT General Hospital, Medical School, National and Kapodistrian University of Athens, Athens, GRC; 4 3rd Department of Orthopaedic Surgery, KAT General Hospital, Medical School, National and Kapodistrian University of Athens, Athens, GRC

**Keywords:** knee function, physiotherapy, preoperative rehabilitation, total knee arthroplasty, osteoarthritis

## Abstract

Purpose: Several studies have shown that patients with severe osteoarthritis (OA) of the knee can reduce their knee pain, improve their quadriceps strength, and improve their functional ability through regular exercise training. The purpose of this study was to investigate the efficacy of a six-week supervised high-intensity preoperative training program on muscle strength, functional performance, and patient-reported outcomes in patients undergoing total knee arthroplasty (TKA).

Methods: Ninety-eight patients scheduled for unilateral TKA for severe OA were allocated to an intervention group (N = 49) who completed a six-week preoperative training program, five days per week prior to surgery, and a control group (N=49) who did not follow any preoperative training program. The Western Ontario and McMaster Universities Osteoarthritis Index (WOMAC), the Physical Functioning Scale of the Short Form-36 questionnaire (SF-36), Knee Injury and Osteoarthritis Outcome Score (KOOS), quadriceps strength, 20-meter walk test, and 30-second chair stand test were assessed at six weeks before surgery (T0), just before surgery (T1), four weeks (T2) and finally 12 weeks (T3) after TKA.

Results: Of 98 patients included in our study, 10 individuals withdrew from the study at different stages. Finally, 44 patients were allocated to the intervention group and 44 patients to the control group. When comparing the changes from baseline to the primary test points at T1, T2, and T3, we found a significant group difference in favor of the intervention group for quadriceps strength (<0.001, 0.001, 0.009), 20-meter walk test (<0.001, 0.023, 0.032), 30-second chair stand test (0.001, <0.001, <0.001) and all patient-reported outcomes WOMAC (<0.001, 0.001, 0.007) except from KOOS that showed significant difference only at T1 (<0.001) at T2 (0.048) but not at T3 (0.087).

Conclusions: Our study demonstrated that a six-week preoperative physiotherapy training program supervised by a physiotherapist before TKA is efficacious for decreasing knee pain, improving knee function, and enhancing daily living activities.

## Introduction

Osteoarthritis (OA) is the most common form of arthritis, predominantly affecting the knees, hips, and hands in the appendicular joints. OA is the most common joint disorder in the US, affecting an estimated 12% of US adults aged 25 to 74 years [1]. Other studies yield a national annual estimate of 30.8 million adults with OA (13.4% of the US adult population) for 2008-2011 [2].

Knee osteoarthritis is a common progressive multifactorial joint disease characterized by chronic pain and functional disability. It is a leading cause of disability and source of societal cost in older adults [3,4]. Moreover, the findings of a recent systemic review and meta-analysis confirmed an increased burden of knee OA, showing a global prevalence of 16% and an incidence of 203 per 10,000 person-years [5].

Older age, female gender, overweight or obesity, knee injury, repetitive use of joints, lack of physical activity, bone density, muscle weakness, and joint laxity all play roles in the development of joint osteoarthritis. Several studies have shown that obesity is the most important risk factor because it not only plays a mechanical role by increasing joint load but additionally plays metabolic and inflammatory roles as a result of the secretion of proinflammatory factors by adipose tissue [6,7].

At the end-stage of the disease, knee replacement surgery is the most common and effective treatment to reduce pain and improve functionality [8]. Total knee arthroplasty (TKA) is a highly successful procedure to alleviate pain and correct instability and deformity of the knee joint. It was reported that the number of TKA procedures would reach 3.48 million in the United States by 2030 [9].

Although TKA surgery is effective for the relief of pain, reduced leg strength may be present for years after surgery [10]. Many authors suggested that reduced lower extremity strength of older adults could lead to a higher incidence of falls and resulted in declines in the ability to perform functional tasks. Indeed, during the first years after TKA, quadriceps strength could decrease by up to 60% and patients have greater functional impairments than age-matched subjects [11,12].

Several studies have shown that patients with severe knee OA can reduce their knee pain, improve their quadriceps strength, and improve their functional ability through regular exercise training [13]. In the last years, preoperative training has been proposed as an effective method for advancing postoperative functional recovery [13-15].

Preoperative physiotherapy and exercise programs (prehabilitation) have been proposed as a potential way to expedite recovery times and optimize functional performance after surgery in patients planning to undergo joint replacement [16]. Various exercise programs are designed to improve leg strength and the ability to perform functional tasks in individuals before TKA. A preoperative training program usually lasts three to eight weeks before surgery and it is mainly focused on increasing quadriceps strength [13,17,18]. Most of the programs could not demonstrate postoperative improvements in functional performance, but there were studies that reported results indicating the efficacy of prehabilitation among TKA patients and supported the theory of prehabilitation [13,19-21]. Moreover, it remains to be clarified whether preoperative training can increase knee muscle strength to a level where it has clinical implications for the postoperative course of patients.

The purpose of this study was to investigate the efficacy of six weeks of a preoperative physiotherapy training program on functional performance, muscle strength, and patient-reported outcomes in patients undergoing TKA. We hypothesized that six weeks of preoperative PRT would be safe and feasible and would improve functional performance, knee extensor and flexor muscle strength, and patient-reported outcomes preoperatively and at four and 12 weeks postoperatively, when compared to controls.

This article was previously presented as a meeting abstract at the 16th Congress of the European Forum for Research in Rehabilitation on September 23-25, 2021.

## Materials and methods

Study design

This was a quasi-experimental trial comprising two parallel groups with repeated measurements. The study was conducted at a single tertiary-care medical center. Baseline testing (T0) occurred six weeks before the participant’s scheduled TKA. All 98 participants were asked to complete the questionnaire package, which consisted of the following: (i) demographic questionnaire, (ii) Western Ontario and McMaster Universities Osteoarthritis Index (WOMAC) score questionnaire, (iii) Knee Injury and Osteoarthritis Outcome Score (KOOS) questionnaire, and (4) Short Form-36 Health Survey (SF-36).

After completing the questionnaires, the participants performed the timed 20-meter flat surface walking test, the timed chair stand test, and the isometric quadriceps extension assessment to assess the functionality of the knee joint (using an isokinetic dynamometer, Humac Norm, Computer Sports Medicine Inc., Massachusetts, USA).

The participants again completed the questionnaires and physical testing at the end of the six-week intervention - just before surgery (T1), as well as at four (T2) and 12 weeks (T3) after their total knee arthroplasty.

Participants

All patients over 60 years old who were diagnosed with advanced idiopathic knee OA (according to the radiological criteria of the American College of Rheumatology Guidelines) and scheduled for unilateral total knee arthroplasty in the Orthopedic Clinic of the University Hospital of Alexandroupolis, Greece from March 2014 until January 2017 were considered candidates for this study and were informed to participate. Exclusion criteria were medical conditions where exercise was contraindicated (i.e., cardiopulmonary comorbidities that precluded modest exercise), diseases that affected their functional performance (suffering from neuromuscular or neurodegenerative conditions), mental diseases, previous hip or knee joint replacement surgery, and if they had severe pain in the controlateral limp that would not allow them to follow any pre- or postoperative interventions.

Eligible participants signed an informed consent document approved by the Research Ethics Committee of the Faculty of Health Sciences, National and Kapodistrian University of Athens (16955: 03/12/2018) prior to participation in the study.

Of the total 234 patients screened, 98 were finally included in our study, and 136 were excluded. Of those, 85 declined to participate and 51 did not meet the inclusion criteria. Patients (N=98) who accepted to participate and fulfilled inclusion criteria were thoroughly informed regarding the six-week preoperative physiotherapy training program and its goals. Patients who accepted to follow a preoperative physiotherapy training program were allocated to the intervention group. On the contrary, patients who refused to follow the preoperative program were allocated to the control group.

**Figure 1:**
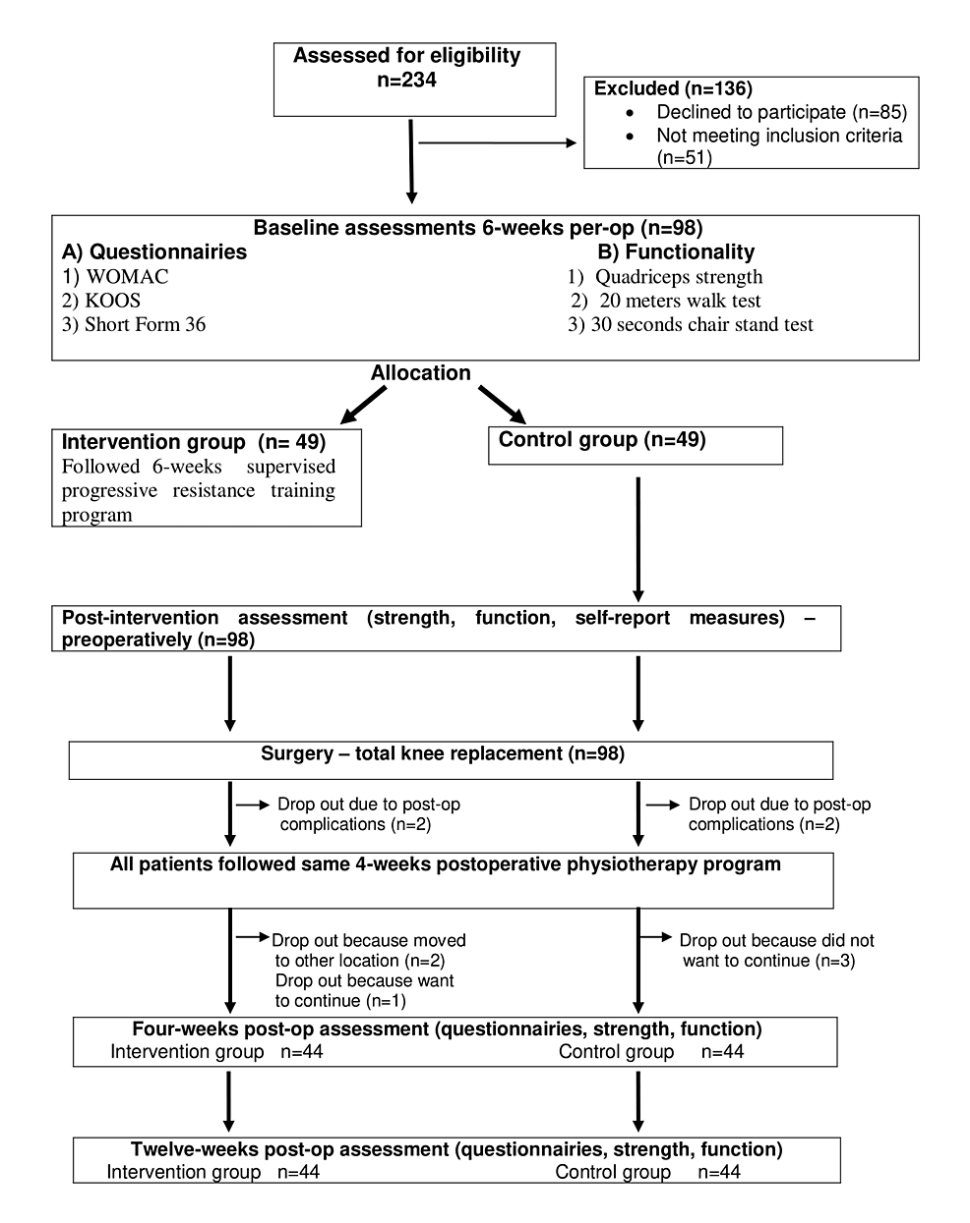
Flow diagram of the progress through the phases of the study

Of those 98 patients, 10 individuals withdrew from the study at different stages for different reasons (four patients had postoperative complications, four patients did not want to continue in the study, and two patients moved to another city). Finally, 44 patients were allocated to the intervention group and 44 patients to the control group (Figure 1).

Subjects assigned (N=44) to the intervention group participated in a six-week preoperative training program after baseline testing measurements were taken. The intervention group performed supervised progressive resistance training five sessions per week for six weeks preoperatively and completed a further five sessions per week for four weeks postoperatively. On the other hand, subjects assigned to the control group had no physical therapy intervention preoperatively, but they followed the same postoperative rehabilitation training program (five sessions/week for four weeks).

Surgical procedures

All patients underwent a TKA, which was implanted with cement with the same standardized preoperative protocol, surgical technique, and performed by the same team of four experienced orthopedic surgeons. In all cases, the posterior cruciate ligament was retained, and the operations were performed with the use of a tourniquet.

Postoperatively, all patients received the same postoperative rehabilitation protocol at the hospital as a part of the usual care treatment. This program was focused on restoring knee ROM, strength, and normal gait. The strength exercises were especially focused on knee extensor strength, starting without external load and progressing by adding a maximum of 2 or 3 kg. This rehabilitation program was performed daily for four weeks, and each session lasted approximately 60 minutes.

Interventions

Patients in the intervention group had a session of preoperative resistance training five days a week for a period of six weeks before TKA. Each session was supervised by a physiotherapist specifically trained in progressive resistance training. The duration of each session was approximately 60 minutes. If a participant missed a training session, it was attempted to substitute the session on an alternative day.

Based on 2009 guidelines of the American College of Sports Medicine, progressive resistance training was defined as a concentric/eccentric muscle contraction against a variable or constant external resistance at a constant or variable velocity, where loading is continuously adjusted to ensure progression [22].

Participants in the intervention group were prescribed a training program that consisted of a 10-minute aerobic warm-up using a treadmill or a stationary bike, followed by a circuit of bilateral lower body exercises (standing calf raise, seated leg press, leg curl, knee extension, knee flexion, and hip abduction) on standard strength training machines (Cybex, Owatonna, MN, USA). Three sets of eight repetitions of each exercise were performed with a rest length of two minutes between sets and exercises. All exercises started at 60% of their one-repetition maximum and increased gradually by increments of 1-2 kg per week, as tolerated, over the course of the six-week intervention.

The load was to be increased only in case more repetitions than prescribed could be made. All sessions concluded with two minutes of stretching exercises for knee extensors, knee flexors, and ankle plantar flexors.

Patients in the control group did not follow any preoperative training program and were instructed to "live as usual" for six weeks preoperatively. Postoperatively, they followed the same progressive training exercise protocol as the intervention group for a period of four weeks.

Outcome measures

This study was designed to evaluate the intervention and its effect on a range of clinically related patient self-assessment and performance-based outcome measures. Several studies concluded that the WOMAC and the Medical Outcomes Study Short-Form 36 (SF-36) could be recommended as primary measures in treatment studies [23]. A systemic review also showed that, overall, regarding patient-reported outcome measures, the KOOS, WOMAC, and SF-36 are the most comprehensively tested tools in this population and are worth considering [24].

For patient-reported outcome measurement, the WOMAC score was available as part of quality control issues [25]. It was applied in the Greek-language version [26]. All patients completed the questionnaire six weeks before the surgical procedure, two or three days before TKA, and four to twelve weeks postoperatively.

The WOMAC is a disease-specific, self-administered questionnaire developed to study patients with hip or knee OA and requires about 10-12 minutes to complete. It has a multidimensional scale made up of 24 items grouped into three dimensions: pain (five items), stiffness (two items), and physical function (17 items). The physical function subscale is more consistent and has stronger test-retest reliability, and the WOMAC Index has generally been shown to exhibit greater or comparable responsiveness to change than other tests [27].

The SF-36 is a valid and reliable instrument for assessing the general health and function of undergoing TKA patients and is a core component of suggested outcome measures for this procedure. We used the Greek version of the SF-36 Health Survey and we evaluated physical functioning. The SF-36 has high test-retest reliability and validity and moderate responsiveness in patients undergoing TKA [28].

The KOOS is a useful scale for evaluating symptoms and functional status related to a knee injury and knee OA. This tool has five subscales, namely: pain, symptoms, daily living, sports and recreational activities, and quality of life-related to the knee. KOOS has high test-retest reliability (ICCs range from 0.74 to 0.97), construct validity, and responsiveness in patients with knee OA [29]. The Greek version of KOOS was used in our study [30].

Quadriceps strength

Muscle strength was measured using an isokinetic dynamometer (HumacNorm, Computer SportsMedicine, Inc., Stoughton, MA). Patients were in a seated position with 900 hip flexions. The anatomical axis of the knee was aligned with the axis of the dynamometer, and the ankle cuff was placed 5 centimeters proximal to the medial malleolus. All patients performed three maximal isometric contractions of the knee extensors at a knee joint angle of 700 (00 = full knee extension) and of the knee flexors at a knee joint angle of 200 with 60 seconds of rest in between [31]. For further analysis, we selected the attempt with the highest peak torque (Nm). Peak torque values were normalized to body weight and reported as Nm/kg.

Dynamometry is considered the gold standard of muscle strength assessment, and dynamometry tests of knee extensor muscles in knee OA have proven reliable with positive ratings for both intra- and inter-tester reliability, responsiveness, and interpretability [32].

20 meters walk test

The 20-meter walk test is frequently used in clinical trials and cohort studies involving individuals with OA, as well as in physical therapy. All participants completed the 20-meter walk test in a 30-meter long, unobstructed hallway. Studies reported that the 20-meter walk test has a high test-re-test reliability among patients with end-stage OA awaiting knee replacements [33].

30 seconds chair stand test

The 30-second chair stand test involves recording the number of stands a person can complete in 30 seconds rather than the amount of time it takes to complete a pre-determined number of repetitions. The participant is seated back straight in the middle of a chair without arms (seat height: 45 cm). The participant is instructed to fully sit between each stand and is encouraged to complete as many full stands as possible within 30 seconds. The investigator recorded the number of stands a participant could complete in 30 seconds. The 30-second chair stand test has excellent test-retest reliability and very good responsiveness and interpretability [34].

The primary outcome measures were quadriceps strength and the WOMAC score, and secondary outcome measures were the 20-meter walk test, 30-second chair stand test, SF-36, and KOOS.

Sample size

The required sample size was calculated using the Power Analysis and Sample Size (PASS 15, NCSS, LLC, Kaysville, Utah) software set for repeated measures. A pilot study was performed in advance of the quasi-experimental trial study with a medium effect size at a power of 80% and a significance level of 0.05. The power calculation indicated that 42 patients should be needed in each study arm to demonstrate a treatment effect. Due to a possible dropout rate of 15%, we planned to include 94 patients in total.

Statistical analysis

Descriptive statistics were calculated with mean±SD for normally distributed data and median and range for non-normally distributed data. The normal distribution of data was checked by q-q plots and histograms. Change scores from baseline to all other test points were compared between the intervention and control groups by repeated-measures analysis of variance, multilevel mixed-effects linear regression. To ensure that the underlying assumptions of the applied statistical model were met, the residuals of the analyses were checked for normal distribution. This check was done for all the performed analyses. A p-value <0.05 was considered statistically significant. SPSS software (SPSS 17. Inc., Chicago, IL) was used for statistical analysis.

## Results

In total, 98 patients were included in this study. Ten individuals withdrew from the study at different stages for different reasons (four patients had postoperative complications, four patients did not want to continue in the study, and two patients moved to another city). None of the patients missed training sessions or were discontinued from the study due to adverse events related to the intervention. Finally, 44 patients were allocated to the intervention group and 44 patients to the control group. Allocation created similar groups at baseline. There was no statistically significant difference between the two groups regarding sex, age, BMI, weight, comorbidities, and WOMAC, SF-36, or KOOS test scores (Table 1).

**Table 1:** Demographic characteristics of participants

	Intervention group (n=44)	Control group (n=44)	P-value
Age (years)	68.7±5.2 (61–81)	68.9±5.4 (60–79)	0.658
Female gender	24 (54.5%)	26 (59.1%)	0.667
No comorbidities	11 (25%)	10 (22.73%)	0.803
BMI (kg/m^2^)	31±4.1 (27–43)	30±3.9 (28–41)	0.851
WOMAC	44.68±11.97	45.32±10.95	0.795
SF-36	34.25±9.48	34.30±9.39	0.947
KOOS	31.98±9.24	34.61±8.85	0.091

Postintervention (just before surgery)

In the intervention group, WOMAC, SF-36, and KOOS scores improved significantly from baseline to the six-week post-intervention preoperative evaluation, whereas they remained unchanged or slightly improved in the control group (Table 2).

**Table 2:** Scores for all the questionnaires

Variable	Assessment time	Mean control group	Mean (± SD) intervention group	P-value
Womac score	Baseline	45.32±10.95	44.68±11.97	0.795
	Pre-op	44.11±11.19	33.75±10.23	<0.001
	4 weeks post-op	30.16±7.44	23.28±5.50	<0.001
	12 weeks post-op	30.52±7.16	24.77±5.89	<0.001
SF-36	Baseline	34.30±9.39	34.25±9.48	0.947
	Pre-op	36.73±8.54	47.20±9.20	<0.001
	4 weeks post-op	48.02±10.08	57.70±7.70	<0.001
	12 weeks post-op	51.52±9.59	56.66±8.76	0.006
KOOS	Baseline	34.61±8.85	31.98±9.24	0.091
	Pre-op	35.93±8.89	41.84±8.68	0.001
	4 weeks post-op	57.68±10.86	58.09±8.39	0.844
	12 weeks post-op	60.66±10.76	60.61±9.80	0.984

These changes resulted in statistically significant changes in WOMAC mean scores (mean difference of 9.7 points, p<0.001), SF-36 mean scores (mean difference of 10.4 points, p 0.001), and KOOS mean scores (mean difference of 9.5 points, p=0.001; Table 3).

**Table 3:** Means and differences between intervention and control group at each test point in WOMAC, SF-36, and KOOS questionnaires

Variable	Assessment time	Mean control group	Mean (± SD) intervention group	P-value
WOMAC	Baseline	45.32±10.95	44.68±11.97	0.795
	Pre-op Test 1 (mean±SD)	44.11±11.19	33.75±10.23	<0.001
	Δ Baseline - Test 1 Mean (95% CI)	−1.21 (−5.90, 3.48)	−10.93 (−15.65, −6.21)	<0.001
	4 weeks post-op (Test 2) (mean±SD)	30.16±7.44	23.28±5.50	<0.001
	Δ Baseline - Test 2 Mean (95% CI)	−15.16 (−19.13, −11.19)	−21.41 (−25.34, −17.45)	0.001
	12 weeks post-op - Test 3 (mean±SD)	30.52±7.16	24.77±5.89	<0.001
	Δ Baseline - Test 3 Mean (95% CI)	−14.80 (−18.72, −10.88)	−19.91 (−23.91, −15.91)	0.007
SF-36	Baseline	34.30±9.39	34.25±9.48	0.947
	Pre-op Test 1 (mean±SD)	36.73±8.54	47.20±9.20	<0.001
	Δ Baseline - Test 1 Mean (95% CI)	2.43 (−1.38, 6.23)	12.95 (8.99, 16.91)	<0.001
	4 weeks post-op (Test 2) (mean±SD)	48.02±10.08	57.70±7.70	<0.001
	Δ Baseline - Test 2 Mean (95% CI)	13.72 (9.59, 17.85)	23.45 (19.79, 27.11)	<0.001
	12 weeks post-op - Test 3 (mean±SD)	51.52±9.59	56.66±8.76	0.006
	Δ Baseline - Test 3 Mean (95% CI)	17.22 (13.20, 21.24)	22.41 (18.54, 26.28)	0.004
KOOS	Baseline	34.61±8.85	31.98±9.24	0.091
	Pre-op Test 1 (mean±SD)	35.93±8.89	41.84±8.68	0.001
	Δ Baseline - Test 1 Mean (95% CI)	1.32 (−2.44, 5.08)	9.86 (6.06, 13.66)	<0.001
	4 weeks post-op (Test 2) (mean±SD)	57.68±10.86	58.09±8.39	0.844
	Δ Baseline - Test 2 Mean (95% CI)	23.07 (18.87, 27.27)	26.11 (22.37, 29.85)	0.048
	12 weeks post-op - Test 3 (mean±SD)	60.66±10.76	60.61±9.80	0.984
	Δ Baseline - Test 3 Mean (95% CI)	26.05 (21.87, 30.23)	28.63 (24.59, 32.67)	0.087

Baseline quadriceps strength scores showed no significant difference between the intervention and control groups (0.88±0.13 vs 0.86±0.12 Nm/kg, p=0.754; Table 4).

**Table 4:** Scores for all physical measures at assessment points

Variable	Assessment time	Mean control group	Mean (± SD) intervention group	P-value
Quadriceps strength	Baseline	0.88±0.07	0.86±0.07	0.754
	Pre-op	0.86±0.09	0.92±0.10	<0.001
	4 weeks post-op	0.70±0.05	0.75±0.08	0.078
	12 weeks post-op	0.87±0.11	0.93±0.11	0.134
20 meters walk test	Baseline	19.00±1,20	18.66±0.97	0.179
	Pre-op	18.96±1.12	18.31±0.82	0.004
	4 weeks post-op	18.19±089	17.60±0.73	0.002
	12 weeks post-op	17.83±0.79	17.18±0.64	<0.001
30-sec chair stand test	Baseline	12.02±1.30	12.43±1.26	0.214
	Pre-op	12.04±1.27	12.82±1.23	0.005
	4 weeks post-op	12.95±1.22	13.93±1.13	0.001
	12 weeks post-op	13.20±1.13	14.36±0.99	<0.001

The preoperative evaluation demonstrated a 17% increase in strength for patients in the intervention group, whereas control group patients showed no significant difference. In comparing the two groups of patients, we found that there was a statistically significant difference between the two groups regarding quadriceps strength at the post-intervention preoperative evaluation (p<0.001) (Table 5).

**Table 5:** Means and differences between intervention and control group at each test point in functional performance and muscle strength outcomes

Variable	Assessment time	Mean control group	Mean (± SD) intervention group	P-value
Quadriceps strength	Baseline	0.88±0.07	0.86±0.07	0.754
	Pre-op Test 1 (mean±SD)	0.86±0.09	0.92±0.10	<0.001
	Δ Baseline - Test 1 mean (95% CI)	0.02 (−0.01, 0.05)	0.06 (0.02, 0.10)	<0.001
	4 weeks post-op (Test 2) (mean±SD)	0.70±0.05	0.75±0.08	0.078
	Δ Baseline - Test 2 mean (95% CI)	−0.18 (−0.15, −0.21)	−0.11 (−0.08, −0.14)	0.001
	12 weeks post-op - Test 3 (mean±SD)	0.87±0.11	0.93±0.11	0.134
	Δ Baseline - Test 3 mean (95% CI)	−0.01 (−0.03, 0.05)	0.07 (0.03, 0.11)	0.009
20-meter walk test	Baseline	19.00±1.20	18.66±0.97	0.179
	Pre-op Test 1 (mean±SD)	18.96±1.12	18.31±0.82	0.004
	Δ Baseline - Test 1 mean (95% CI)	−0.04 (−0.45, 0.53)	−0.35 (−0.72, 0.03)	<0.001
	4 weeks post-op (Test 2) (mean±SD)	18.19±0.89	17.64±0.73	0.002
	Δ Baseline - Test 2 mean (95% CI)	0.81 (0.36, 1.26)	1.02 (0.66, 1.38)	0.023
	12 weeks post-op - Test 3 (mean±SD)	17.83±0.79	17.28±0.64	<0.001
	Δ Baseline - Test 3 mean (95% CI)	1.17 (0.74–1.60)	1.38 (1.03, 1.73)	0.032
30-sec chair stand test	Baseline	12.02±1.30	12.43±1.26	0.214
	Pre-op Test 1 (mean±SD)	12.04±1.27	12.82±1.23	0.005
	Δ Baseline –Test 1 mean (95% CI)	0.02 (−0.52, 0.54)	0.39 (0.14, 0.92)	0.001
	4 weeks post-op (Test 2) (mean±SD)	12.95±1.22	13.93±1.13	0.001
	Δ Baseline - Test 2 mean (95% CI)	0.93 (0.40, 1.96)	1.50 (0.99, 2.00)	<0.001
	12 weeks post-op - Test 3 (mean±SD)	13.20±1.13	14.36±0.99	<0.001
	Δ Baseline –Test 3 mean (95% CI)	1.18 (0.66, 1.70)	1.93 (1.45, 2.41)	<0.001

Regarding the other functional tasks, our statistical analysis showed that both the 20-meter walk test and 30-second chair stand test scores improved significantly from baseline to post-intervention, just before surgery evaluation, whereas they remained stable in the control group (Table 4), which resulted in a statistically significant difference for both the 20-meter walk test (p<0.001) and the 30-second chair stand test (p=0.001) scores between the two groups of patients (Table 5).

Postoperative period

Evaluation of all patients four weeks postoperatively (T2) demonstrated that maximal torque of the knee extensors showed no statistically significant difference between the two groups of patients (0.70±0.21 vs 0.72±0.25, p=0.454) and it was decreased from T1 (preoperative evaluation) to T2 in both groups of patients (control group 21.7% vs intervention group 23.8%; Table 4). The comparison of the two groups showed that there was a statistically significant difference in maximal torque of the knee extensors between the intervention and control groups for baseline evaluation T0 to T2 evaluation (p<0.001; Table 5).

Although there was a statistically significant difference in WOMAC, SF-36, and KOOS score changes from baseline to the four-week postoperative evaluation (p=0.001, p=0.001, and p=0.048, respectively) between the two groups of patients (Table 3).

Regarding the other functional tasks, our statistical analysis showed that for both the 20-meter walk test and the 30-second chair stand test scores, there was a statistically significant difference between the intervention and control group when we compared baseline T0 scores and postoperative T2 scores (p=0.023 and p<0.001, respectively; Table 5).

Finally, evaluation of all patients 12 weeks postoperatively (T3) demonstrated that changes in maximal torque of the knee extensors showed a statistically significant difference between the two groups of patients (0.07 vs −0.01, p=0.009; Table 5). There was an 8.9% increase in strength for patients in the intervention group from baseline, whereas control group patients showed no significant difference at week 12 (Table 5).

Regarding the other functional tasks, our statistical analysis showed that for both the 20-meter walk test and the 30-second chair stand test scores, there was a statistically significant difference between the intervention and control groups when we compared baseline T0 scores and postoperative T3 scores (p=0.032 and p<0.001, respectively; Table 5).

Although there was a significant difference in WOMAC and SF-36 score changes from baseline to the 12-week postoperative evaluation (p=0.007 and p<0.004, respectively) between the two groups of patients, the KOOS score changes did not show any significant difference (p=0.087; Table 3).

## Discussion

This study describes the effectiveness of a preoperative exercise on the postoperative recovery of a TKA population in a Greek setting. In this study, we evaluated the effect of a six-week preoperative exercise intervention on self-assessed and performance-based measures of functional status before and after TKA, using a quasi-experimental trial design. We found that a six-week supervised preoperative progressive resistance training program resulted in improvements in functional performance, muscle strength, and patient-reported outcomes in older patients undergoing TKA.

The present study demonstrated a significant effect of the prehabilitation program on quadriceps muscle strength when compared to the control group, which was achieved without increasing pain. Significant effects of preoperative progressive resistance training were also noticed in patient-related functional performance and health-related quality of life, except for the KOOS scale.

The significant improvements in our study could be attributed to higher training intensity and the application of unilateral versus bilateral training. We also demonstrated that intensive strength training is feasible for the majority of the patients awaiting TKA since no patients dropped out of the intervention group because of the training intensity.

The WOMAC and SF-36 scores improved over time in both groups. This could be expected due to the benefits of the surgery per se. However, greater benefits were shown in favor of the intervention group in all evaluations for WOMAC and SF-36 (T1<0.001, T2<0.001, T3<0.001 and T1<0.001, T2<0.001, T3=0.006, respectively).

The ability to sit and stand up from a chair and the ability to walk are activities of daily living and predictors of mobility and functional capacity. Several studies found no improvement for both activities after preoperative training [13,14,17,18,35]. On the contrary, we demonstrated progressively improved performance in both functional tasks among the intervention group, a finding that is in accordance with recently published studies [36].

Patients in the intervention group significantly increased their quadriceps strength preoperatively (T1) compared to their baseline evaluation T0 (p<0.001). Our study also found a statistically significant difference between the intervention and control groups regarding quadriceps strength at the post-intervention/just before surgery T1 evaluation (p<0.001).

These findings suggest that patients in the intervention group present a significant improvement in quadriceps strength compared to control group patients who did not follow any preoperative training intervention.

Another interesting finding of our study is that patients in the intervention group showed a significant improvement in their functional performance preoperatively compared to control group patients. This improvement was statistically significant for both patient-reported outcome measurements (WOMAC, SF-36, and KOOS questionnaires) and functional tasks (20-meter walk test and 30-second chair stand test), suggesting that patients with severe knee OA benefit from high-intensity training.

We also found that patients following a six-week preoperative training program presented a statistically significant improvement in all functional performance measurements, patient-reported outcome, strength measurement, and functional tasks at four (T2) and 12 (T3) weeks postoperative evaluation compared to the control group. These findings are in accordance with recently published articles reporting significant improvements in quadriceps strength and functional performance in patients following a preoperative training program [36,37].

Relationships between quadriceps strength and physical performance

Increases in quadriceps strength and performance of functional tasks before TKA surgery may result in improved postoperative recovery because the preoperative performance of functional tasks has been shown to be a predictor of postoperative performance of functional tasks [37].

Previous literature failed to report a positive effect of preoperative programs on maximal strength variables after TKA [13,14,17,18,38,39]. In terms of study design, one of the major limitations of most RCTs was the very small sample sizes they used. The numbers of participants in some studies were as low as 10 patients per treatment arm [17,18,20].

The results from these studies were therefore not adequately powered. McKay et al. found that patients in the intervention group showed no advantage over the control participants after surgery, which indicated that the simple strengthening exercises in this intervention did not train neuromuscular activation to the extent necessary to overcome this deficit [17].

On the contrary, recent studies have shown that high-intensity strength training during the preoperative period can improve postoperative functional performance, muscle strength, and patient-reported outcomes [35,36]. Skoffer et al. found that a preoperative progressive resistance program can improve postoperative functional performance and muscle strength, but they did not detect any significant improvements in patient-reported outcomes [35]. Another randomized controlled study showed that high-intensity strength training during the preoperative period reduces pain and improves lower limb muscle strength and functional task performance before surgery, resulting in faster physical and functional recovery after TKA [36].

Most interventions involved multi-modal physiotherapy, encompassing a combination of different types of exercises - warm-up, aerobic exercise, resistance training, flexibility training, proprioceptive training, and practicing functional tasks - with or without patient education. As there is little evidence as to which types of exercise are most effective in bringing about improvements in postoperative outcomes, a combination approach may dilute the impact of more effective elements.

Juhl et al. conducted a systematic review of 48 studies investigating the effects of exercise programs on pain and patient-reported disability in knee OA. The authors concluded that exercise programs focusing on a single type of exercise showed higher efficacy in reducing pain and patient-reported disability than programs that included several types of exercise with different goals within the same session. Optimal exercise programs for knee OA should have one aim and focus on improving aerobic capacity, quadriceps muscle strength, or lower extremity performance. This result needs to be taken into consideration when planning exercise interventions before and after TKA [40].

In a systematic review and meta-analysis, Wallis and Taylor concluded that there is low-to-moderate evidence from mostly small randomized controlled trials demonstrating that preoperative exercise interventions may reduce pain in TKA patients [41]. Another systematic review and meta-analysis identified seven studies of preoperative rehabilitation before TKA. The results of this review indicated that preoperative rehabilitation likely had no true treatment effect on WOMAC scores or range of motion, with the exception of a trend towards a shortening of the length of the hospital stay [42].

However, it is noteworthy that none of the studies included in those systemic reviews have applied to high-intensity resistance training programs. Additionally, the majority of published studies on the effects of a prehabilitation intervention used prehabilitation programs of shorter duration (three-four weeks), and these studies often show no or little effect of prehabilitation.

Proper training intensity and frequency seem to be important to achieve optimal gains in muscle strength and could be the main reason why previous research on preoperative training did not significantly improve treatment success. Overall, the present study found that a six-week preoperative training program showed important clinical benefits in terms of improvements in many physical characteristics known to be important for the daily functioning of patients after TKA.

Strengths and limitations

Our study is a quasi-experimental study with a relatively large number of patients. The training protocol was designed according to the recommendations of the American College of Sports Medicine, integrating higher training intensity and volume during training sessions, for example, the number of sets per muscle group and the total time of training for five times/week.

Additionally, a specially trained physiotherapist supervised all patients during their training sessions, ensuring that they continually trained close to the maximum of their capability and followed the plan of progression.

This study has several limitations that should be kept in mind when interpreting the results. Although this study is a quasi-experimental study and we allocated patients to two groups based on their hospital track number and not on clinical or radiological features, it is very difficult to blind patients and train physiotherapists to an exercise intervention because a placebo intervention is easily recognized by both patients and physiotherapists. Another limitation is that the preoperative exercise group received additional attention from the physiotherapists, which may have impacted the preoperative patient-reported outcomes scores. Despite that the intervention was effective, it is likely that education about self-management, exercise, and coping strategies together with the training program could have provided even better outcome scores. Finally, it should be taken into account that the present results may not be extended beyond 3 months after TKA. Further randomized controlled studies with a large number of patients could be followed up for 12-24 months postoperatively in order to examine the long-term effects of preoperative training programs.

## Conclusions

Our study supports that a six-week preoperative physiotherapy training program supervised by a physiotherapist before TKA is efficacious for decreasing knee pain, improving knee function, and enhancing daily living activities in patients with severe knee osteoarthritis. The present training program could be considered and used by specialists to speed up recovery early after TKA, which, together with proper post-operative training, could lead to even further benefits. Further well-designed randomized controlled studies with a large number of patients are needed to replicate our findings before clinical recommendations are made. Additionally, large sample studies with many different intervention arms could also determine the proper training intensity, duration, and frequency in order to achieve optimal gains in muscle strength.
